# Autobiographical memory retrieval in the context of self-schema updating: Does specific recall have power?

**DOI:** 10.3758/s13421-025-01785-y

**Published:** 2025-08-27

**Authors:** Noboru Matsumoto

**Affiliations:** Division of Psychology, Faculty of Arts, Shinshu University, 3-1-1 Asahi, Matsumoto, Nagano 390-8621 Japan

**Keywords:** Self-schema, Belief updating, Autobiographical memory specificity, Depression, Memory accessibility

## Abstract

Self-schemas are formed from the common elements of past experiences. In clinical contexts, maladaptive negative self-schemas are associated with resistance to therapeutic interventions and prospectively predict a worse course of depression. One possible way to update self-schemas is to recall specific autobiographical events that support or contradict current self-schemas. This study investigated whether retrieving specific autobiographical memories facilitates self-schema updating more effectively than retrieving general memories or no memories. Additionally, the study explored whether depressive symptom severity was associated with memory accessibility biases. Undergraduate students (N = 101) completed an autobiographical memory task where they recalled specific memories in which they or their friend behaved consistently or inconsistently with adjective cues (e.g., competent, inferior). Participants rated how well these traits applied to themselves (i.e., assessment of self-schema) before and after the autobiographical memory retrieval. Results showed that retrieving memories in which participants behaved consistently or inconsistently with their traits led to changes in self-schema in the corresponding direction, and this effect was more pronounced when specific memories were recalled. Furthermore, shorter retrieval latencies for self-inconsistent memories were associated with greater changes in self-schema. Depressive symptom severity was associated with shorter retrieval latencies only in the condition where memories were inconsistent with positive self-traits and in the condition where they were consistent with negative self-traits. These findings highlight the utility of specific autobiographical recall in self-schema updating and suggest that interventions targeting autobiographical memory specificity may effectively address negative self-schemas, particularly in individuals with dysphoria.

**Supplementary Information:**

The online version contains supplementary material available at 10.3758/s13421-025-01785-y.

## Introduction

Studies have shown that self-schemas, or internal representations of the self, are closely related to shaping identity and personality (McAdams et al., 2021). Adaptive self-schemas support psychological well-being, whereas negative self-schemas are a transdiagnostic feature of mental disorders that lead to the persistence and exacerbation of depression (Disner et al., 2011; Dozois & Beck, 2008; Kube, 2023). Self-schemas guide one’s future and predict behavior, and are adjusted in response to the external environment and experiences (Markus, 1977). Recent studies have highlighted the dynamic nature of self-schemas, showing that they can be updated to integrate new information derived from life events (Matsumoto et al., 2023a; Moscovitch et al., 2023). Previous studies have demonstrated that events consistent with prior schemas further strengthen those schemas, whereas inconsistent events weaken them (Kahan, 2016; Kube & Rozenkrantz, 2021; Sharot et al., 2023). Furthermore, depressed individuals underestimate the weighting (i.e., accuracy) of new information and experience that contradict negative prior schemas, making such schemas less likely to be updated (Kube, 2023).

While these studies have explored the influence of new experiences on self-schemas, the role of autobiographical memory retrieval in updating self-schemas has not been systematically examined since Wagenaar (1992). Research on autobiographical memory has demonstrated that it has a critical role in organizing a person’s self, identity, and life story (Conway & Pleydell-Pearce, 2000; McAdams, 2011; Prebble et al., 2013; Singer & Blagov, 2004). In particular, self-defining memories, which are vivid, emotionally intense, repeatedly recalled, thematically linked to similar memories, and focused on enduring concerns or unresolved conflicts (Singer & Salovey, 1993), are known to form an individual’s identity. Previous studies have demonstrated that recalling positive self-defining memories leads to high self-esteem (Çili & Stopa, 2015; Hallford & Mellor, 2016). However, the specific mechanisms by which more extensive autobiographical memory retrieval, not limited to self-defined memories, influences self-schemas remain underexplored, with only a few recent studies investigating this issue (Moscovitch et al., 2024; Sheldon et al., 2024).

Autobiographical memory retrieval increased the accessibility to self traits (Charlesworth et al., 2016; Sakaki, 2007; for a review, see Rathbone & Moulin, 2024). This finding suggests that the retrieval process references self-schemas, which may often occur when individuals reflect on their self-concept, such as by asking themselves whether they are competent, kind, or reliable (e.g., “Am I competent?”). In this process, they may retrieve autobiographical memories that either support self-schema (e.g., “I finished writing this manuscript”) or contradict it (e.g., “A manuscript I submitted was rejected”; Byrne et al., 1999; Hitchcock et al., 2017). Episodic memory plays a crucial role in forming and updating abstract concepts and schemas, as demonstrated in classic studies on concept learning (Bruner et al., 1956; Griggs & Cox, 1982). For instance, confidence in a category requires retaining and retrieving both positive and negative instances from past experiences (Bruner et al., 1956). Based on these findings that highlight the importance of episodic memory in forming and updating schema, retrieving autobiographical memories that support or challenge one’s self-schema can serve as evidence for self-schema updating. Most autobiographical memories are forgotten or lack self-relevance (Brown, 2023). Nevertheless, recognizing their connection to the self can provide meaningful evidence for self-schema updating.

Researchers widely agree that self-schemas and autobiographical memory are connected through a gradation of specificity and abstraction, underpinned by distinct but cooperative neural basis. Although terminology and distinctions vary between fields, at the very least, autobiographical memory can be categorized into three levels: specific memories, general memories (e.g., repeated events or gist memories), and self-schemas (Gilboa & Moscovitch, 2021; Grilli & Verfaellie, 2014; Renoult et al., 2012; Williams et al., 2007). General memories are formed by similar specific memories (Barsalou, 1988; Williams & Dritschel, 1992). When general memories are further abstracted and detached from experiences, they become self-schemas (Ghosh & Gilboa, 2014; Markus, 1977, 1983). Therefore, specific memories can be viewed as the minimal elements that consist of self-schemas.

In the context of therapeutics, researchers emphasize the significance of referencing specific events and reinterpreting their meanings (Marsh et al., 2023; Speer et al., 2021). Cognitive behavior therapy includes a program that aims to update self-schema by helping patients recognize positive autobiographical memories that contradict negative self-schemas (Beck, 2021). In addition, to reinforce adaptive self-schemas, which are not necessarily overly positive, patients are encouraged to gather evidence from future events and memories. Cognitive behavior therapy assumes that retrieving memories consistent with self-schemas reinforces those schemas, whereas retrieving memories inconsistent with self-schemas weakens them.

For several reasons, focusing on specific memories may be more effective than focusing on general memories and directly modifying self-schema when updating them. Firstly, self-schemas and general memories are more difficult to modify directly because they are highly abstract. In contrast, specific memories are easier to update and reinterpret. Sensory and perceptual details in specific events, though emotionally neutral, may be misinterpreted negatively. Thus, reinterpreting such sensory details can provide positive meanings to specific memories (Speer et al., 2021) and buffer against negative self-schemas.

Secondly, given that self-schemas are formed through the accumulation of similar specific memories, working with specific memories can be a fundamental technique for updating self-schema. Although general memories (e.g., “My boss often praises me”) can be generated, they remain fragile unless grounded in specific instances (e.g., “My boss praised me when my manuscript was accepted”). Without such a foundation, general memories may carry limited weight and have less impact on updating self-schemas.

Thirdly, compared to general memories, specific memories hold greater personal significance, evoke a stronger sense of re-experience, and elicit intense emotions when retrieved (Addis et al., 2004; Levine et al., 2004). Thus, retrieving specific memories may have a more significant influence on self-schemas. These considerations suggest that specific memory retrieval contributes more to updating self-schema than general or no memory retrieval. However, to the best of the author’s knowledge, this hypothesis has not yet been tested empirically.

Given that accessing (specific) memories plays a vital role in self-schema updating, reduced accessibility to these memories may hinder the updating process. Depression is characterized by reduced autobiographical memory specificity, notably diminished access to positive specific memories and increased access to negative general memories (Claúdio et al., 2012; Dalgleish & Werner-Seidler, 2014; Gupta & Kar, 2012). Hitchcock et al. (2017) extended Klein et al.’s (2002) scope hypothesis to the context of depression. Klein et al. posit that retrieving specific memories inconsistent with current self-schemas serves as a boundary condition for self-schema evaluation. According to Klein et al., specific memories inconsistent with a given trait are retrieved more quickly than those consistent with it immediately after judging how well a trait applies to the self. Klein et al. (2002) argued that when people evaluate their self-traits (e.g., “How kind am I?”), they can rely on schematic knowledge without needing to access specific memories that support those traits. This schematic knowledge reflects people’s average self-perception across situations, without taking contextual variations into account. However, the generalizability of self-traits requires referencing specific memories inconsistent with those traits and serves as boundary conditions (e.g., I did not give up my seat on the bus for a pregnant woman because I was tired). Therefore, in self-trait judgments, both schematic knowledge and specific memories that are inconsistent with self-traits are activated. The latter are primed in this process, resulting in shorter retrieval latency.

Hitchcock et al. (2017) reported that, after evaluating how well a trait applies to the self, individuals with depression had faster access to negative autobiographical memories inconsistent with the positive self. In contrast, healthy controls had faster access to positive autobiographical memories inconsistent with the negative self. These findings suggest that individuals with depression have weaker boundaries for their negative self-schema, leading to the generalization of these schemas, whereas they have strict boundary conditions for their positive self-schema. Summarizing these findings in the context of evaluating one’s self-schema suggests that difficulties in retrieving positive memories lead to maintaining and generalizing negative self-schemas, and increased accessibility to negative memories prevents the formation of positive self-schemas in depression. These compelling hypotheses must be tested within the framework of schema updating.

This study therefore investigated whether retrieving autobiographical memories leads to self-schema updating, with a focus on the roles of memory specificity and retrieval latency. By examining changes in self-schema before and after retrieving memories, the study aimed to identify the mechanisms underlying self-schema updating and explore their implications for individuals with depressive symptoms. First, participants quickly evaluated their self-schema (e.g., happy, incompetent) for either positive or negative traits, without time for memory retrieval. Next, they recalled specific memories in which they behaved consistently or inconsistently with the presented traits. As a control condition, they also recalled specific memories in which their friend behaved consistently or inconsistently with the presented traits. Finally, participants re-evaluated their self-schema after memory retrieval.

The study tested three chief hypotheses. The first set of hypotheses addressed the relationship between memory retrieval and self-schema updating. We predicted that retrieving memories of one’s own behavior consistent with a given trait would strengthen self-schema, whereas retrieving inconsistent memories would weaken self-schema (Hypothesis 1-1). For comparison, retrieving memories of others’ behavior was expected to show a smaller effect than retrieving memories of one’s own behavior. We also predicted that the degree of self-schema updating would be greater when retrieving specific memories than when retrieving general memories (Hypothesis 1-2).

The second set of hypotheses concerned the relationship between depressive symptom severity and memory retrieval latency. We predicted that depressive symptom severity would be associated with faster retrieval latency (Hypothesis 2-1) when retrieving memories inconsistent with the positive self. Furthermore, it was predicted that depressive symptom severity would be associated with slower retrieval latency (Hypothesis 2-2) when retrieving memories inconsistent with the negative self. These hypotheses on retrieval latency replicate those tested by Hitchcock et al. (2017). In addition, the study predicted that depressive symptom severity would be associated with faster retrieval latency (Hypothesis 2-3) for memories consistent with the negative self. These predictions align with previous findings that individuals with depression are more likely to activate and directly access general memories in response to negative cues (Hallford & Matsumoto, 2022; Matsumoto et al., 2020, 2023a, b). Finally, since Hitchcock et al. (2017) reported no association between depression and retrieval latency for memories consistent with the positive self, we examined this association in an exploratory way.

The third set of hypotheses focused on the relationship between depressive symptom severity and memory specificity. The study predicted that depressive symptom severity would be associated with more specific memories (Hypothesis 3-1) when retrieving memories inconsistent with the positive self. Conversely, it was expected that depressive symptom severity would be associated with fewer specific memories (Hypothesis 3-2) when retrieving memories inconsistent with the negative self. Additionally, based on previous findings as mentioned above (Hallford & Matsumoto, 2022; Matsumoto et al., 2020, 2023a, b), it was predicted that depressive symptom severity would be associated with more general memories (Hypothesis 3-3) when retrieving memories consistent with the negative self. Similar to the retrieval latency, we also explored the relationship between depressive symptom severity and memory specificity in memories consistent with the positive self condition.

Finally, this study aimed to explore the associations between depressive symptom severity and self-schema updating. We planned to explore mediation relationships regarding whether depressive symptom severity is associated with retrieval latency and memory specificity abnormalities, which may, in turn, lead to biased self-schema updating. These analyses were planned only if an association were found between depressive symptom severity and self-schema updating, and Hypothesis 2 or 3 was supported. This study was preregistered, and all materials are available on the Open Science Framework (10.17605/OSF.IO/UFGN6).

## Methods

### Participants and sample size determination

Japanese undergraduate students from Shinshu University were recruited via the SONA system. The target sample size was set at 100 to ensure stable estimation of fixed and random effects in a linear mixed model (Maas & Hox, 2005). Recruitment was stopped once 100 participants had registered, resulting in a final sample size of N = 101 (43 males, 57 females, one other; *M*_age_ = 18.71, *SD* = 1.09 years).

### Beck Depression Inventory–Second Edition (BDI-II)

The Japanese version of the Beck Depression Inventory–Second Edition (BDI-II; Beck et al., 1996; Kojima & Furukawa, 2003) was used to assess the participants’ depressive symptom severity. The BDI-II consists of 21 items and required answers using a 4-point scale ranging from 0 to 3. Internal consistency in this study was acceptable (Cronbach’s α =.75).

### Autobiographical memory task

The autobiographical memory task consisted of 48 trials and included three factors: Self/Other-reference (2: Self/Other) × Cue valence (2: Positive/Negative) × Consistency (2: Consistent/Inconsistent), with six trials per condition. The study used 24 positive and 24 negative adjectives^1^ as cue words, which were randomly assigned across conditions. These adjectives were selected based on data from word characteristics pooled in the author’s laboratory (N = 79). Positive and negative cues did not differ in terms of imaginability or familiarity, although they did differ in emotional valence. Moreover, there was no significant difference in the number of characters between positive and negative cues in word length (*M*s = 3.25 vs. 3.33; *t* = 0.21, *p* =.83). In each trial, participants were asked to rate how well the adjective displayed on the screen applied to themselves within 4 s using a 9-point Likert scale, ranging from 1 (*Not at all*) to 9 (*Very much*). They were then instructed to recall a specific memory in which either they (Self-reference condition) or a friend (Other-reference condition) behaved in a way consistent or inconsistent with the adjective. Specifically, participants were instructed as follows.

*“In some trials, we would like you to think of and talk about a past event in which you behaved in a way that is consistent with or not consistent with a word presented on the screen. In other trials, we would like you to think of and talk about a past event in which your friends behaved in a way that is consistent with or not consistent with a word presented on the screen. Events can be recent, such as something that happened yesterday or last week, or something that happened a long time ago. They can be important events or trivial events. However, the events you recall should be specific events, meaning they lasted less than 1 day and occurred at a particular time and place.”*

**Fig. 1:**
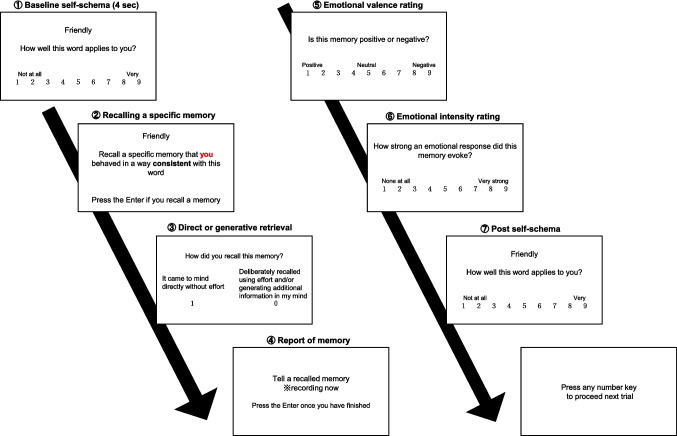
Overview of the experiment. Data from direct and generative retrieval judgments was not used in this paper

In the other-reference condition, participants were allowed to recall events involving any friend or acquaintance. Retrieval latency was defined as the time between cue presentation and pressing the Enter key. Afterward, participants rated the retrieval process (“*How did you recall this memory*?”) by selecting 1 (*It came to mind directly without effor*t) or 0 (*I deliberately recalled it using effort and/or by generating additional information in my mind*). Then, they reported the memory orally. All responses were recorded using a voice recorder and later transcribed by research assistants. Following this, participants rated the emotional valence (*Is this memory positive or negative?* 1: positive, 5: neutral, 9: negative) and the emotional intensity (*How strong an emotional response did this memory evoke?* 1: none at all, 9: very strong) on a 9-point Likert scale. If participants recalled no memory within the time limit, the trial proceeded without any responses. Finally, participants re-evaluated how well the adjective applied to themselves on the same 9-point scale. There was no time limit for this task. To familiarize participants with the sequence of the task, they first conducted two practice trials (cue words: 友好的[friendly] and 賢い[smart]) before starting the main trial. In the practice trials, the experimenter confirmed that participants had a full understanding of the task. Figure 1 shows the overview of the experiment.

After the experiment, two independent raters categorized participants’ recalled memories into five types after the experiment. This classification followed standard procedures used in memory-specificity studies (e.g., Crane et al., 2007; Raes et al., 2007). The five categories included the following:

- Specific memory, defined as an event that occurred at a particular time and place and lasted no more than 1 day (e.g., “I went to the Melbourne Museum yesterday”);
- Categoric memory, referring to summaries of similar events (e.g., “I often go shopping at Woolworths”);
- Extended memory, referring to an event that lasted more than 1 day (e.g., “I visited Australia as a guest researcher for 2 months”);
- Semantic association, involving semantic knowledge that is not an event (e.g., “I love the city of Melbourne”); and
- Omission, including cases where no memory was recalled or the response was otherwise inappropriate.

The inter-rater agreement was good (Cohen’s κ =.78), and any discrepancies in classification were resolved through discussions between the raters.

### Procedure

The experiment was conducted individually in a face-to-face setting. After providing informed consent, participants completed several questionnaires, including RRS (Ruminative Responses Scale), LEIDS-R (Leiden Index of Depression Sensitivity-Revised), B-PNI (Brief Pathological Narcissism Inventory), and CRT (Cognitive Reflection Test) in addition to BDI-II. These questionnaires were intended for an exploratory analysis and are not reported in this study, although the data are publicly available. After completing the questionnaires, participants performed the autobiographical memory task. They received 1,000 JPY as compensation for their participation upon completion of the task. This study was conducted with the approval of the ethics committee of the Faculty of Arts, Shinshu University (Approval ID: 24002).

### Statistical models

All analyses were conducted using R statistics. Given the hierarchical structure of the data, with trials nested within participants, linear mixed models were employed using the lme4 package. The study observed 4,848 trials, which were nested within 101 participants. A random intercept and fixed slope model was used by including random slopes for trial order when feasible (i.e., when singular fit errors did not occur). Before the analysis, the retrieval latency was log transformed due to positive skewness in the distribution (skewness = 0.643). Changes in self-schemas were calculated by subtracting pre-recall ratings from post-recall ratings, such that positive values reflected increases in self-schema endorsement. Dichotomous explanatory variables were developed as follows: cue valence (positive = 0.5, negative = –0.5), consistency (consistent = 0.5, inconsistent = –0.5), and self/other-reference (self = 0.5, other = –0.5).

To examine Hypothesis 1-1, cue valence, consistency, self-reference, and their interactions were entered into a model predicting changes in self-schema to examine Hypothesis 1-1. Here the other-reference condition served as a control comparison for the self-reference condition. Trials were excluded if participants failed to respond to the pre-recall self-schema rating within 4 s or failed to recall any memory (i.e., semantic associations or omissions). This procedure resulted in 2,937 trials nested within 101 participants for the analysis. A significant two-way interaction was expected between consistency and self/other-reference, as well as significant simple main effects of self-reference under both consistent and inconsistent conditions relative to the other-reference condition.

For Hypothesis 1-2, the analysis was restricted to trials in the self-reference condition to simplify the model. Memory specificity (specific memory vs. general memory) was entered as a third factor instead of self/other-reference. Following the memory specificity literature (e.g., Barry, Hallford, Hitchcock et al. 2021a, Barry, Hallford, Takano et al. 2021b), general memories encompass categoric and extended memories. Consequently, 1,523 trials nested within 101 participants were used in this analysis. The study expected to find a significant two-way interaction between consistency and memory specificity, as well as significant simple main effects of specific memory recall on self-schema changes in both consistent and inconsistent conditions.

To test Hypotheses 2-1, 2-2, and 2-3, the retrieval latency was modeled as a function of cue valence, consistency, self/other-reference, depressive symptom severity, and their interactions, after excluding trials involving semantic associations or omissions. This analysis included 100 participants, as one questionnaire response was missing, yielding 3,493 trials for analysis. We expected to find a significant four-way interaction and significant simple main effects of depressive symptom severity in positive self-inconsistent condition (Hypothesis 2-1), negative self-inconsistent condition (Hypothesis 2-2), and negative self-consistent condition (Hypothesis 2-3).

To test Hypotheses 3-1, 3-2, and 3-3, dichotomous variables were developed indicating (a) whether a specific memory was recalled (specific memory = 1; all other responses including omissions = 0) and (b) whether a categoric memory was recalled (categoric memory = 1; all other responses = 0). Here, 4,800 trials nested within 100 participants were used for the analysis. Cue valence, consistency, self-reference, depressive symptom severity, and their interactions were entered as predictors of specific memory recall (for Hypotheses 3-1 and 3-2) and categoric memory recall (for Hypothesis 3-3). We expected to find a significant four-way interaction and significant simple main effects of depressive symptom severity in positive self-inconsistent condition (Hypothesis 3-1), in negative self-inconsistent condition (Hypothesis 3-2), and in negative self-consistent condition (Hypothesis 3-3).

### Exploratory analysis

A 2-1-1 mediation analysis was planned, in which depressive symptom severity would be related to memory specificity (coded as 0 or 1 for each trial) or retrieval latency, which in turn would predict changes in self-schema. This analysis was planned only if a relationship were observed between depressive symptom severity and self-schema updating, and either Hypothesis 2 or Hypothesis 3 was supported. Therefore, we first examined whether depressive symptom severity was associated with self-schema updating by entering cue valence, consistency, depressive symptom severity, and their interactions into a model predicting self-schema updating. In addition, we also examined whether retrieval latency was related to self-schema updating.

## Results

### Descriptive statistics and correlations

Table 1 presents the number of responses across conditions defined by cue valence, self/other-reference, and consistency. The mean memory specificity was 0.37 ± 0.18; when omissions were excluded, the mean was 0.50 ± 0.22. Omissions were observed in 24.6% of all trials. The frequency of semantic associations was very low, accounting for only 2.5% of responses. A chi-square test was conducted to examine whether the frequency of omissions varied as a function of cue valence, self/other-reference, and consistency, yielding a significant three-way interaction (*p* <.001). Post hoc comparisons using the *emmeans* package in R showed that omissions were significantly more frequent in the other-reference condition than in the self-reference condition for negative consistent condition (*z.ratio* = 4.05, p <.001) and positive inconsistent condition (*z.ratio* = 2.66, p =.039). Comparing cue valence revealed more omissions for negative cues than positive cues in the other-reference consistent condition *(z.ratio* = 4.75, p <.001) and other-reference inconsistent condition (*z.ratio* = 2.77, p =.029). Furthermore, more omissions were observed for inconsistent trials than for consistent trials in the negative self-reference condition (*z.ratio* = 2.86, p =.022) and positive other-reference condition (*z.ratio* = 5.45, p <.001).

**Table 1:** Descriptive statistics for the number of responses in each condition

		Positive				Negative			
		Consistent		Inconsistent		Consistent		Inconsistent	
		Frequency	Proportion (%)	Frequency	Proportion (%)	Frequency	Proportion (%)	Frequency	Proportion (%)
Self-reference	Specific	234	38.61	206	33.99	226	37.29	198	32.67
	Categoric	204	33.66	191	31.52	211	34.82	186	30.69
	Extended	36	5.94	48	7.92	42	6.93	42	6.93
	Semantic	10	1.65	10	1.65	12	1.98	17	2.81
	Omission	122	20.13	151	24.92	115	18.98	163	26.90
Other-reference	Specific	269	44.39	207	34.16	228	37.62	236	38.94
	Categoric	191	31.52	140	23.10	147	24.26	157	25.91
	Extended	20	3.30	44	7.26	32	5.28	40	6.60
	Semantic	22	3.63	14	2.31	13	2.15	24	3.96
	Omission	104	17.16	201	33.17	186	30.69	149	24.59

Table 2. presents the retrieval latency across conditions. Table 3 shows participant-level Pearson correlations and trial-level multilevel correlations (Kenny & la Voie, 1985) among depressive symptom severity, baseline self-schema ratings (for positive and negative cues), retrieval latency, and memory specificity. Memory specificity was calculated based on whether the response was classified as specific or not (i.e., categoric, extended, semantic, or omission). Depressive symptom severity and memory specificity were used only for participant-level correlations. Results showed that higher levels of depression were associated with lower ratings of positive words (*r* = -.35, *p* =.005) and higher ratings of negative words (*r* =.59, *p* <.001). At the trial level, greater self-schema ratings were associated with shorter retrieval latencies (*r* = -.05, *p* =.006), although the effect size was quite small. The mean BDI-II score was 12.53 ± 7.09 (range: 1–37), which is slightly below the cutoff for mild depressive symptoms (≥ 14).

**Table 2.** Descriptive statistics for retrieval latency across trials in each condtion

	Positive				Negative			
	Consistent		Inconsistent		Consistent		Inconsistent	
	Mean	SD	Mean	SD	Mean	SD	Mean	SD
Self-reference	10.64	6.97	11.95	7.18	10.77	6.93	12.22	6.79
Other-reference	11.25	6.90	13.19	7.03	12.12	7.15	12.66	6.84

**Table 3:** Intra- and inter-correlations between depressive symptom severity (BDI- II), baseline self-schema, retrieval latency, and memory specificity

	BDI-II	Self-Schema Baseline	Retrieval Latency
*In All Trials*			
Self-Schema Baseline	.18	**.06** ***	-.05 **
Retrieval Latency	-.20	-.21	**.22** ***
Memory Specificity	.03	.21	.17
*For Positive Cues*			
Self-Schema Baseline	-.35 **	**.21** ***	-.04
Retrieval Latency	-.21	-.04	**.27** ***
Memory Specificity	.06	.23	.12
*For Negative Cues*			
Self-Schema Baseline	.59 ***	**.17** ***	-.09 ***
Retrieval Latency	-.17	-.12	**.20** ***
Memory Specificity	.01	.05	.21

*** *p* <.001, ** *p* <.01, * p <.05

Note. Trial-level correlations are indicated by underlining. Bold values indicate inter-class correlations. The other values represent participant-level correlations

### Hypothesis 1-1

**Fig. 2:**
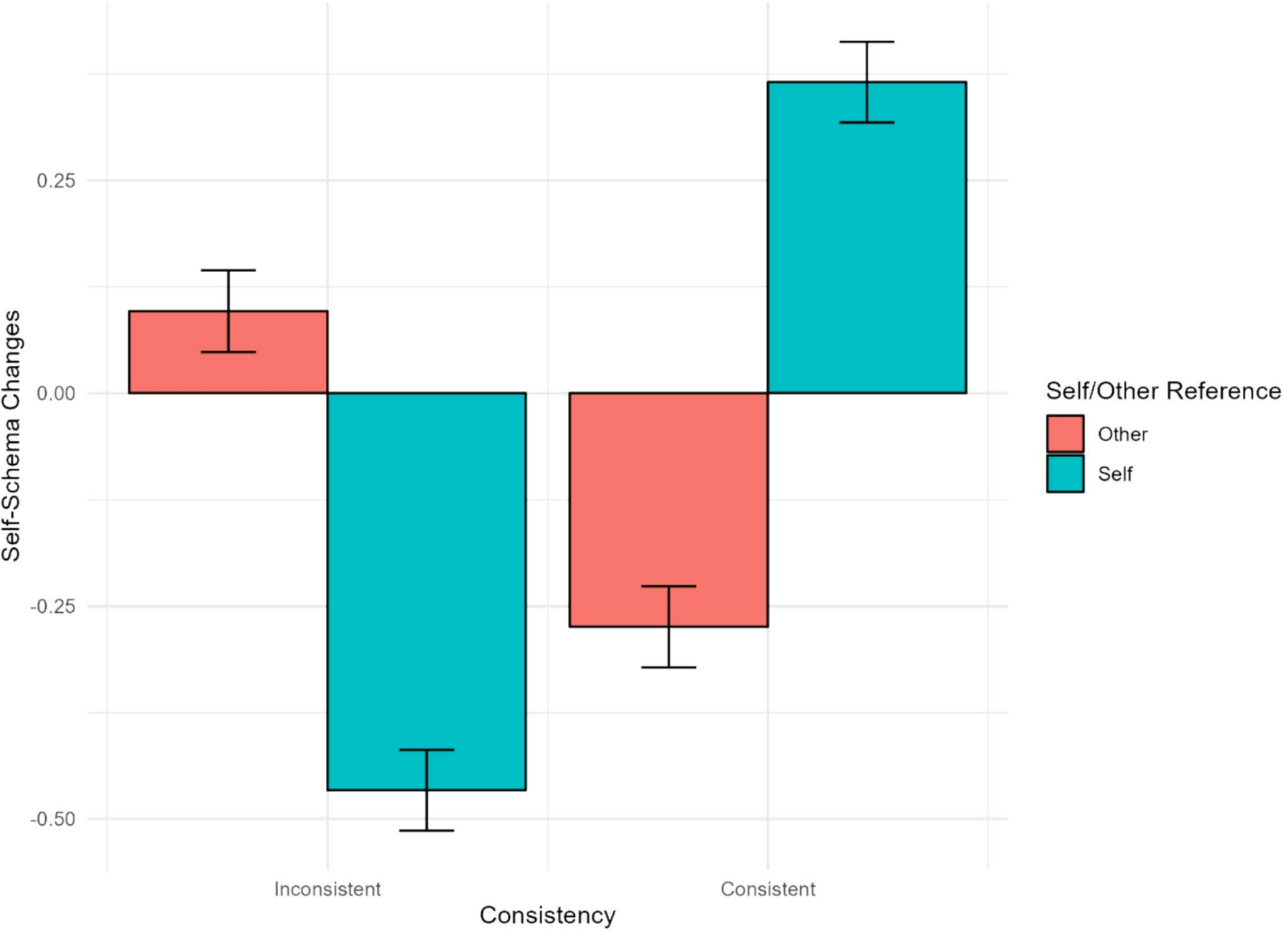
Interaction between self- and other-reference and consistency in self-schema updating

Hypothesis 1-1 examined the effect of self-referent versus other-referent memories, either consistent or inconsistent with the presented traits, on updating self-schemas. As expected, the interaction between self/other-reference and consistency was significant (*γ* = 1.61, *95% CI* [1.41, 1.80], *t* = 16.46, *p* <.001; see Table 4). Compared to the other-reference condition, in the self-reference condition, consistent memory recall led to increases in self-schema ratings (*γ* = 0.84, *95% CI* [0.71, 0.98], t = 12.26, *p* <.001), whereas inconsistent memory recall led to decreases in self-schema ratings (*γ* = −0.76, *95% CI* [−0.90, −0.63], *t* = −11.03, *p* <.001; see Fig. 2). In the self-reference condition, consistent memory recall led to increases in self-schema ratings compared to inconsistent memory recall (*γ* = 1.18, *95% CI* [1.05, 1.32], *t* = −17.22, *p* <.001). In the other-reference condition, consistent memory recall led to more decreases in self-schema ratings than inconsistent memory recall (*γ* = −0.42, *95% CI* [−0.56, −0.29], *t* = −6.10, *p* <.001).

**Table 4:** Linear mixed model for changes in self-schemas (Hypothesis 1-1)

*Predictors*	*Estimates*	*95%CI*	*p*
(Intercept)	−0.09	−0.17 – −0.00	**.046**
Cue valence	0.10	−0.04 – 0.24	.146
Consistency	−0.45	−0.59 – −0.31	**<.001**
Self/Other	0.01	−0.09 – 0.10	.884
Cue valence * Consistency	0.27	−0.01 – 0.54	.059
Cue valence * Self/Other	−0.14	−0.33 – 0.05	.150
Consistency * Self/Other	1.61	1.41–1.80	**<.001**
Cue valence * Consistency * Self/Other	−0.27	−0.66 – 0.11	.160
**Random Effects**			
σ^2^	1.72		
^τ^00 ParticipantID	0.05		
^τ^00TrialID	0.01		
ICC	0.03		
^N^ParticipantID	101		
^N^TrialID	48		
Observations	2937		
Marginal R^2^/Conditional R^2^	0.102/0.130

### Hypothesis 1-2

**Fig. 3:**
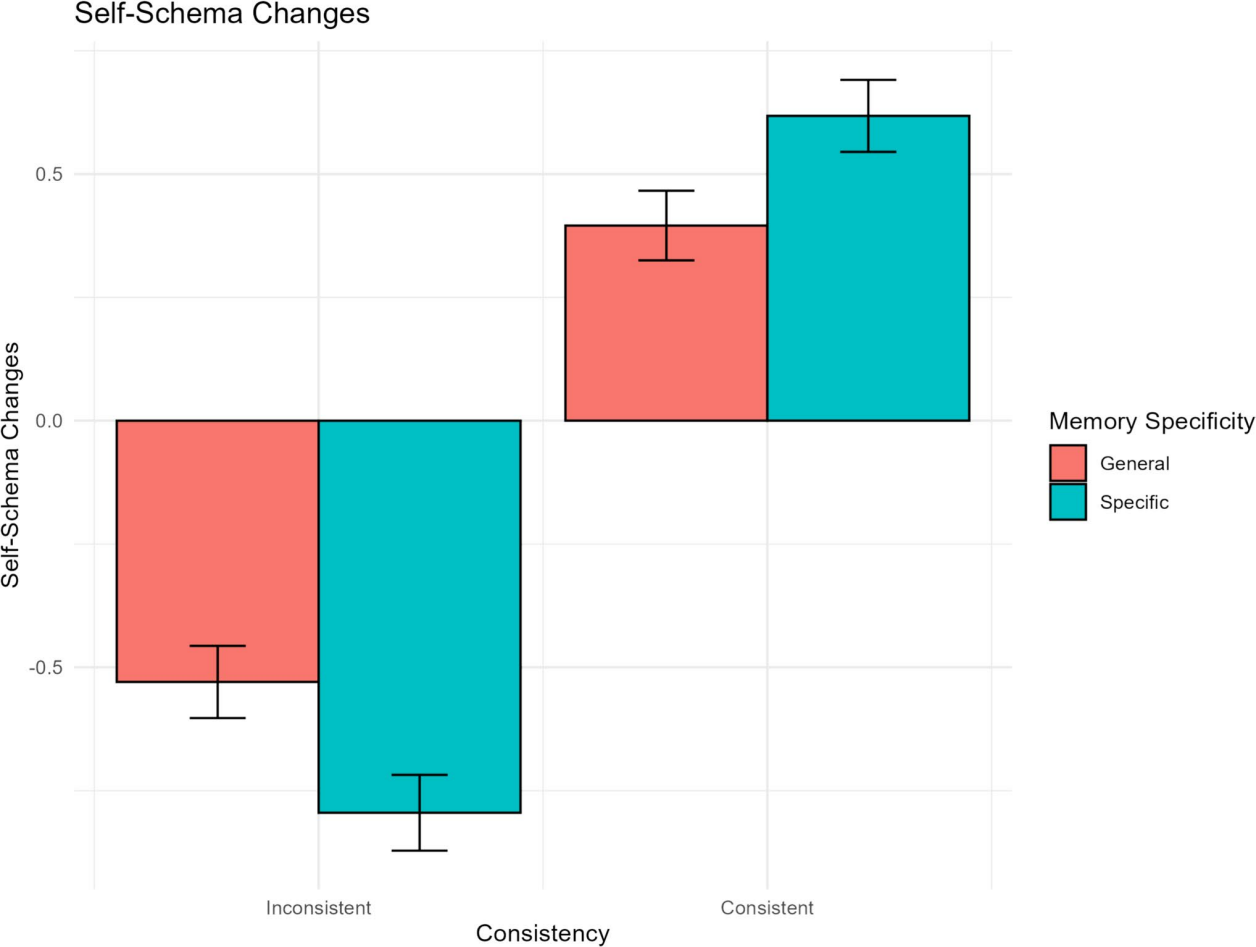
Interaction between Self-Consistency and Memory Specificity in Self-Schema Updating

Hypothesis 1-2 tested whether the degree of self-schema updating differed between specific and general memory recall. A significant interaction was found between consistency and memory specificity (*γ* = 0.49, *95% CI* [0.21, 0.77], *t* = 3.45, *p* <.001; see Table 5). Compared to general memory recall, specific memory recall in the self-consistent condition led to greater increases in self-schema ratings (*γ* = 0.24, *95% CI* [0.04, 0.44], *t* = 2.33, *p* =.02), whereas specific memory recall in the self-inconsistent condition led to larger decreases in self-schema ratings (*γ* = −0.26, *95% CI* [−0.46, −0.06], *t* = −2.51, *p* =.012; see Fig. 3). Within specific memory recall, consistent memories led to larger increases in self-schema ratings than inconsistent memories (*γ* = 1.40, *95% CI* [1.21, 1.60], *t* = 14.01, *p* <.001). Moreover, within general memory recall, consistent memories also led to more increases in self-schema ratings than inconsistent memories (*γ* = 0.91, *95% CI* [0.71, 1.11], *t* = 9.03, *p* <.001). These simple main effects remained significant even after controlling for emotional intensity (*p*s <.02).

**Table 5:** Linear mixed model for changes in self-schemas (Hypothesis 1-2)

*Predictors*	*Estimates*	*95%CI*	*p*
(Intercept)	−0.08	−0.15 – 0.00	.051
Cue valence	−0.04	−0.18 – 0.10	.554
Consistency	1.17	1.03–1.31	**<.001**
Specific/General	−0.02	−0.16 – 0.12	.776
Cue valence * Consistency	−0.01	−0.29 – 0.26	.920
Cue valence * Specific/General	−0.04	−0.32 – 0.24	.768
Consistency * Specific/General	0.49	0.21–0.77	**<.001**
Cue valence * Consistency * Specific/General	−0.23	−0.79 – 0.33	.413
**Random Effects**			
σ^2^	1.90		
^τ^00ParticipantID	0.03		
ICC	0.01		
^N^ParticipantID	101		
Observations	1523		
Marginal R^2^/Conditional R^2^	0.155/0.167

### Hypotheses 2-1, 2-2, and 2-3

**Fig. 4:**
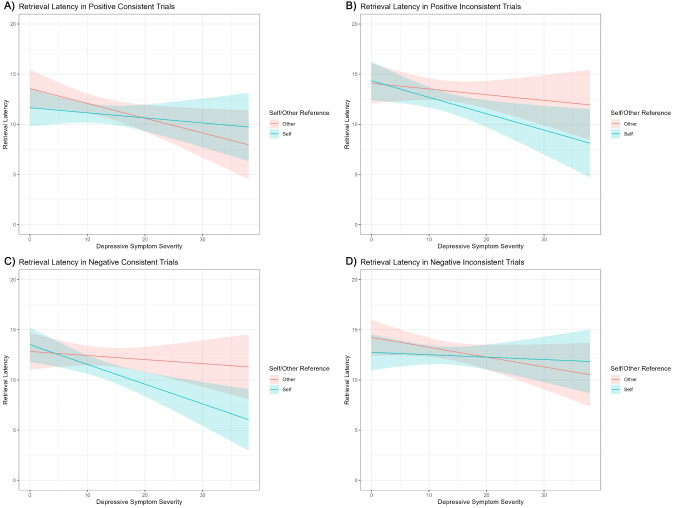
Predicted values of retrieval latency as a function of depressive symptom severity, consistency, and self/other-reference. (A) Retrieval latency in positive consistent trials. (**B**) Retrieval latency in positive inconsistent trials (Hypothesis 2-1). (C) Retrieval latency in negative consistent trials (Hypothesis 2-3). (D) Retrieval latency in negative inconsistent trials (Hypothesis 2-2)

Hypothesis 2 examined whether depressive symptom severity predicts retrieval latency under different experimental conditions. Consistent with expectations, the retrieval latency model revealed a significant four-way interaction (see Table 6.). As planned, post hoc tests were conducted to examine the effects of depressive symptom severity in self-inconsistent memory for positive cues (Hypothesis 2-1), self-inconsistent memory for negative cues (Hypothesis 2-2), and self-consistent memory for negative cues (Hypothesis 2-3). In the self-inconsistent memory condition for positive cues, the simple main effect of depression was significant (*γ* = −0.02, *95% CI* [−0.03, −0.01], *t* = −3.37, *p* <.001), indicating that higher depression rating was associated with shorter retrieval latencies (see Fig. 4B). In the self-inconsistent memory condition for negative cues, the simple main effect of depression was not significant (*γ* = −0.00, *95% CI* [−0.02, 0.01], *t* = −0.75, *p* =.45; see Fig. 4D). In the self-consistent memory condition for negative cues, the simple main effect of depression was evident (*γ* = −0.03, *95% CI* [−0.04, −0.01], *t* = −4.35, *p* <.001), indicating that higher depression rating is associated with shorter retrieval latencies (see Fig. 4C). Thus, Hypotheses 2-1 and 2-3 were supported. However, Hypothesis 2-2 was not.

**Table 6.** Linear mixed model for retrieval latency

*Predictors*	*Estimates*	*95%CI*	*p*
(Intercept)	2.46	2.32–2.59	**<.001**
Cue valence	−0.02	−0.09 – 0.06	.701
Self/Other	−0.06	−0.14 – 0.02	.134
Consistency	−0.13	−0.21 – −0.05	**.001**
BDI-II	−0.01	−0.02 – −0.00	**.020**
Cue valence * Self/Other	−0.12	−0.27 – 0.04	.147
Cue valence * Consistency	−0.16	−0.31 – −0.00	**.045**
Self/Other * Consistency	−0.01	−0.17 – 0.15	.901
Cue valence * BDI-II	0.00	−0.01 – 0.00	.582
Self/Other * BDI-II	0.00	−0.01 – 0.00	.130
Consistency * BDI-II	0.00	−0.01 – 0.00	.492
Cue valence * Self/Other * Consistency	−0.52	−0.83 – −0.21	**.001**
Cue valence * Self/Other * BDI-II	0.01	−0.00 – 0.02	.124
Cue valence * Consistency * BDI-II	0.01	0.00–0.02	**.045**
Self/Other * Consistency * BDI-II	0.00	−0.01 – 0.01	.857
Cue valence * Self/Other * Consistency * BDI-II	0.05	0.03–0.07	**<.001**
**Random Effects**			
σ^2^	0.33		
^τ^00ParticipantID	0.11		
^τ^00TrialID	0.00		
ICC	0.25		
^N^ParticipantID	100		
^N^TrialID	48		
Observations	3493		
Marginal R^2^/Conditional R^2^	0.044/0.283

The exploratory analyses revealed no significant effect of depression on retrieval latency in self-consistent memory for positive cues (*γ* = −0.00, *95% CI* [−0.01, 0.01], *t* = −0.51, *p* =.61; see Fig. 4A). The simple main effect of depression was significant in the other-consistent memory condition for positive cues (*γ* = −0.02, *95% CI* [−0.03, −0.00], *t* = −2.77, *p* =.006), indicating that higher depression rates are associated with shorter retrieval latencies (also shown in Fig. 4A). No significant effects of depression were observed in the remaining combinations of cue valence and consistency in other-reference trials (*p*s >.10).

### Hypotheses 3-1, 3-2, and 3-3

Hypothesis 3 examined whether depressive symptom severity affects the recall of specific memories and categoric memories under several conditions. The generalized linear mixed model for specific memory responses (vs. other types of responses) revealed a significant main effect of consistency (*OR* = 1.46*, 95% CI* [1.13, 1.89], *p* =.004) and a significant interaction between cue valence and consistency (*OR* = 1.74, *95% CI* [1.04, 2.91], *p* =.035; see Table S1 in the Online Supplemental Material). These findings indicate that self- and other-consistent trials elicited more specific memories than inconsistent trials, with this effect being more pronounced for positive cues (*z* = 3.53, *p* <.001). Contrary to expectations, the four-way interaction was not significant (*OR* = 0.96, *95% CI* [0.89, 1.03], p =.28).

The generalized linear mixed model for categoric memory responses (vs. other responses) showed significant main effects of self/other-reference (*OR* = 1.65, *95% CI* [1.26, 2.15], *p* <.001) and consistency (*OR* = 1.35, *95% CI* [1.03, 1.76], *p* =.029). However, none of the interaction terms reached significance (*all p*s >.07; see Table S2, Online Supplemental Material), failing to support any of the predictions in Hypothesis 3.

### Exploratory analysis

**Fig. 5:**
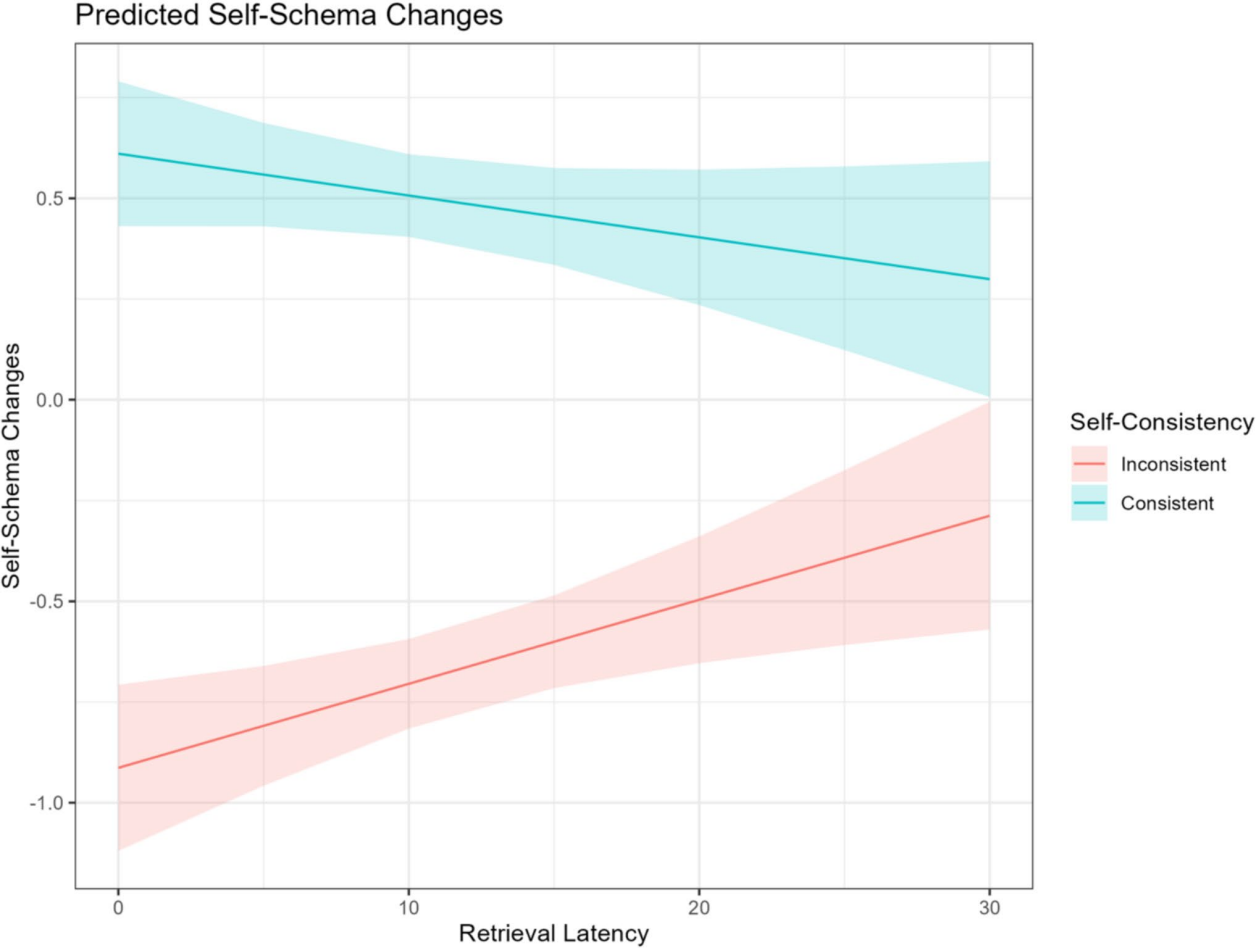
Self-schema changes as a function of retrieval latency in self-consistent and self-inconsistent memory recall conditions

For changes in self-schema, we found no main effect of depression or significant interactions involving depression (see Table S3, Online Supplemental Material); therefore, we did not conduct the planned mediation analysis. Instead, the study examined the association between retrieval latency and changes in self-schema. The retrieval latency, cue valence, consistency, and their interactions were entered into the model, with a focus on self-reference trials (see Table S4, Online Supplemental Material). Results indicated a significant interaction between retrieval latency and consistency (*γ* = −0.33, *95% CI* [−0.53, −0.13], *t* = −3.26, *p* =.001). Post hoc analysis indicated that faster retrieval latency was associated with greater reductions in self-schema ratings in inconsistent trials (*γ* = 0.28, *95% CI* [0.13, 0.43], *t* = 3.62, *p* <.001; see Fig. 5). This finding extends the scope hypothesis (Klein et al., 2002) to the context of self-schema updating, independent of depressive symptom severity.

## Discussion

This study examined whether (specific) autobiographical memory recall contributes to changes in self-schema. In addition, we examined whether high levels of depressive symptoms were associated with retrieval latency and memory specificity, and whether these factors, in turn, led to abnormalities in self-schema updating. To this end, we asked participants to recall specific memories of themselves or others behaving in a manner congruent or incongruent with a given trait and to evaluate their self-schemas before and after recalling these memories.

In support of Hypothesis 1-1, retrieving memories of one’s own behavior consistent with a given trait reinforced self-schemas, whereas recalling inconsistent memories weakened them. This effect was observed regardless of the cue valence. Furthermore, in support of Hypothesis 1-2, self-schema updating was more pronounced following specific memory recall than general memory recall. This effect was not attributable to the emotional response evoked during memory retrieval. These results suggest that retrieving specific autobiographical memories is useful for challenging self-schemas in a positive or negative direction, and that these changes may not be simply due to the emotional response induced by memory retrieval. Previous research has documented the dynamic nature of self-concept (Conway, 2005), as even the retrieval of a single autobiographical memory can activate self-traits (Charlesworth et al., 2016; D’Argembeau & Garcia Jimenez, 2024). Consistent with these notions, our results suggest that autobiographical memory retrieval contributes to the dynamic updating of self-schemas.

Recent research on schema updating has attracted much attention (Kube & Rozenkrantz, 2021; Sharot et al., 2023), and these studies suggest that ongoing and new experiences can update existing schemas. Our findings extend this body of work by demonstrating that autobiographical memory retrieval can update self-schemas. These results align with recent findings by Moscovitch et al. (2024), who showed that positive autobiographical recall contributed to updating negative schemas in individuals with social anxiety. Taken together, this growing body of evidence supports the notion that autobiographical memory retrieval may be a promising approach for modifying negative self-schemas. Because self-schemas are constructed from specific sets of autobiographical memories (Dalgleish & Hitchcock, 2023), the retrieval and integration of memories that are either consistent or inconsistent with existing self-schemas is expected to have a meaningful impact on those schemas.

Studies have shown that memory specificity training (MeST) improves mental health outcomes (Barry et al., 2019; Raes et al., 2009). This type of training typically involves repeatedly practicing the recall of specific memories, as reduced memory specificity has been shown to predict a poorer course of depression (Hallford et al., 2021), as well as impairments in social problem solving and episodic future thinking (Williams et al., 2007). Based on our findings, the therapeutic effects of MeST may partly arise from the updating of self-schema through specific autobiographical recall. The working mechanisms underlying MeST are still debated (Barry, Hallford, Hitchcock et al., 2021a, Barry, Hallford, Takano et al., 2021b), and researchers are increasingly interested in optimizing this therapeutic effect. This study’s findings suggest that providing a context in which to recall specific memories inconsistent with current negative self-schemas, rather than simply retrieving positive specific memories, may lead to greater changes in those schemas.

Cognitive reappraisal, a core component of cognitive behavior therapy, aims to reduce negative emotions by identifying specific events and updating the dysfunctional cognitions and schemas that underlie these emotions into more adaptive alternatives (Beck, 2021). Clinicians have long recognized that focusing on specific, detailed events, rather than general or nonspecific events, is more likely to promote cognitive change (Marsh et al., 2023; Speer et al., 2021). One meta-analysis found that autobiographical memory specificity does not boost the treatment effects of mindfulness-based cognitive therapy or standard cognitive therapy (Hitchcock et al., 2022). However, the ability to recall specific memories that provide evidence to disconfirm a negative self-schema may be different from memory specificity in general. Future studies should focus more on autobiographical memory specificity in the context of self-schema updating.

Another implication of the finding that specific memory retrieval leads to more effective self-schema updating than general memory retrieval relates to the function of reduced memory specificity. According to the functional avoidance hypothesis, reduced memory specificity works as an emotion regulation strategy (Williams et al., 2007). Avoiding the retrieval of negative specific memories may not only prevent the experience of negative emotions associated with those memories but also protect an individual from negative impacts on self-schemas. Our findings could extend the functional avoidance hypothesis to include its influence on self-schema maintenance and change.

In line with Hypotheses 2-1 and 2-3, depressive symptom severity was associated with faster retrieval of inconsistent memories for positive self and consistent memories for negative self. This pattern may reflect the heightened accessibility of negative memories frequently observed in individuals with depression. In particular, the faster retrieval of inconsistent memories for positive self supports prior findings by Hitchcock et al. (2017), suggesting that individuals with depression may impose stricter boundary conditions when evaluating positive self-schemas.

Interestingly, depression-related changes in retrieval latency did not predict self-schema updating. Instead, faster retrieval of memories inconsistent with the presented traits was generally associated with weakened self-schemas, regardless of depressive symptom severity. These results extend the hypothesis regarding boundary conditions in memory retrieval to the context of self-schema updating, but they do not support its exclusive application to individuals with dysphoria.

Contrary to Hypothesis 2-2, this study did not find a relationship between depressive symptom severity and slower retrieval of inconsistent memories for negative self, which contrasts with the results of Hitchcock et al. (2017). One possible explanation for this discrepancy is the difference in sample populations: Hitchcock et al. studied individuals with a clinical diagnosis of depression, whereas the current study recruited nonclinical undergraduate students. Dysphoric participants in this study may have held more positive self-schemas than clinically depressed individuals, and therefore positive memories may have served as counter-examples to negative self-views more readily, comparable to healthy individuals. Indeed, in the present study, the correlation between depressive symptom severity and evaluations of positive self was weaker than the correlation with evaluations of negative self (see Table 3). Anhedonia, a core diagnostic criterion of depression, is associated with a diminished positive self-concept (Dunn et al., 2009) and predicts the diagnosis of depression in the future (Stringaris et al., 2015). Thus, slow retrieval of inconsistent memories for the negative self may be more characteristic of clinically depressed individuals who have weakened positive self-schemas than of individuals with subclinical dysphoria.

The results did not support Hypotheses 3, as depressive symptom severity was not associated with memory specificity in the context of self-schema updating. This finding diverges from the robust body of evidence supporting a link between depression and reduced memory specificity (Barry, Hallford, & Takano, 2021b; Liu et al., 2013; Ono et al., 2016). One possible reason for this null finding could be the use of the Autobiographical Memory Test–Traditional Instruction (AMT-TI; Williams & Broadbent, 1986) in this study, which emphasizes that participants recall specific memories as a goal. Prior research has shown that this format can obscure associations between depressive symptoms and reduced memory specificity in nonclinical samples (Debeer et al., 2009). In contrast, it has been demonstrated that the AMT-MI (Minimal Instruction), which allows participants to recall any type of memory, identifies such associations more clearly (Debeer et al., 2009). Assessing initial responses using the AMT-MI may help clarify whether memory specificity mediates the relationship between depressive symptoms and self-schema updating. Another possible explanation is that the self/other-reference and consistent/inconsistent instructions in this study may have functioned as retrieval cues, facilitating access to specific memories and thereby attenuating the relationship between reduced memory specificity and depressive symptoms. Given that autobiographical memory retrieval depends on effective cue generation (Conway & Pleydell-Pearce, 2000; Uzer et al., 2012), these structured instructions may have aided specific memory retrieval even for participants with dysphoria.

This study found no evidence of specific difficulties in self-schema updating related to depression by retrieving autobiographical memories. As this study was conducted with nonclinical students, it remains possible that clinical depression involves abnormalities in self-schema updating not observed in the current sample. Nonetheless, our findings suggest that the use of specific autobiographical recall to promote changes in self-schema may be equally effective for individuals with or without dysphoria. Negative self-schemas and their high interconnectedness have been identified as vulnerability factors for depression (Dozois & Rnic, 2015). Updating self-schemas through autobiographical memory retrieval may represent a promising strategy for the prevention of depression. Our findings support the development and implementation of training programs that enhance the retrieval of positive autobiographical memories as a preventative intervention for clinical depression (e.g., Hallford et al., 2024; Hitchcock et al., 2016).

### Limitations and future directions

Specific limitations constrain the findings of this study. First, the study sample consisted solely of non-clinical Japanese undergraduate students, which may limit the generalizability of the results to other populations. It remains necessary to confirm whether self-schema changes through memory retrieval can also be observed in individuals with clinical depression. Second, we controlled several aspects of the positive and negative cue words, including imageability, familiarity, and number of characters, but not frequency,^2^ which could have influenced the results related to cue valence. Third, some of the observed self-schema changes could have been affected by demand characteristics, although comparisons between specific and general memory recall are less likely to be affected, as participants were unaware of the memory-specificity hypothesis. Future studies may address this limitation by using measures of implicit self-schema. Fourth, this study did not examine the long-term stability of self-schema changes. Future research should investigate whether self-schema updating through memory retrieval results in lasting changes or merely temporary fluctuations. Fifth, we treated self-schemas as individual items rather than as a structured cluster (Dozois & Rnic, 2015). Therefore, it remains unclear whether updating one self-schema influences other interconnected self-schemas. Given that self-schemas comprise multiple dimensions (Dapp et al., 2023; Linville, 1985), it would be valuable to investigate how updates to specific schemas propagate within the broader self-schema network. Finally, searching for multiple specific events, rather than a single event, may be more effective for self-schema updating. This is because inductive reasoning, essential for forming generalized schemas, relies on identifying commonalities across multiple experiences (Albrecht et al., 2021; Barsalou et al., 1998; Hayes et al., 2010). Future research should explore strategies that facilitate effective self-schema updating through autobiographical memory retrieval, while considering the underlying reasoning process.

## Conclusion

This study showed that specific autobiographical recall effectively updates self-schemas. We also found that high accessibility (i.e., shorter retrieval latency) to memories inconsistent with the target self-schema leads to greater changes in the self-schema. Furthermore, individuals with dysphoria exhibited heightened accessibility to memories inconsistent with the positive self and consistent with the negative self. These results underscore the potential of psychological interventions aimed at improving memory specificity (Barry et al., 2019; Raes et al., 2009) and enhancing positive memories (Hallford et al., 2024; Hitchcock et al., 2016), thereby facilitating self-schema updating. Future research should examine whether the act of recalling memories alone is sufficient or whether framing memory retrieval within the context of explicit self-schema evaluation leads to more effective self-schema updating. This study contributes to the growing literature on the relationship between the self and autobiographical memory, and sheds new light on autobiographical memory retrieval in the context of self-schema updating.

## Supplementary Information

Below is the link to the electronic supplementary material.

Supplementary file1 (DOCX 38 KB)

Supplementary file2 (DOCX 31 KB)

## Data Availability

The data and the analytic codes are available on the Open Science Framework (10.17605/OSF.IO/WVXG3).

## Footnotes

1 As the experiment was conducted using Japanese language, we indicated the original cue words and their English translations in brackets. Positive cues were 勇敢 (brave), 愛される (beloved), 有能 (competent), 思いやりがある (compassionate), 頼もしい (dependable), 幸せ (happy), 面白い (funny), 温厚 (warm), 人懐っこい (friendly), 親切 (kind), 好奇心旺盛 (curious), 一生懸命 (diligent), 誠実 (sincere), 陽気 (lively), 社交的 (sociable), 魅力的 (charming), 努力家 (hardworking), 慕われている (respected), 元気 (energetic), 明るい (cheerful), 義理堅い (loyal), 知的 (intellectual), 楽観的 (optimistic), and 几帳面 (methodical). Negative cues were 劣った (inferior), 不器用 (clumsy), 孤独 (lonely), 内気 (shy), 見捨てられた (abandoned), 依存的 (dependent), 未熟 (immature), 怒りっぽい (hot-tempered), しつこい (persistent), 傲慢 (arrogant), 臆病 (cowardly), 無価値 (worthless), 神経質 (nervous), 無気力 (lethargic), 優柔不断 (indecisive), 不安定 (unstable), 卑怯 (dirty), 怠惰 (lazy), 自分勝手 (selfish), おせっかい (meddlesome), 大雑把 (rough), 空気が読めない (can't take a hint), 心配性 (worrying), and 役立たず (useless).

2 In response to a reviewer's comment, I conducted a post hoc attempt to address this issue, but unfortunately, frequency information for many of the cue words used in the autobiographical memory task was not available in any Japanese corpora due to a characteristic feature of the Japanese language.

## Code availability

The analytic code is available on the Open Science Framework.
