# Supplementary material for: Autobiographical memory retrieval in the context of self-schema updating: Does specific recall have power?
Source: Mem Cognit. 2025 Aug 27;54(3):865–82. doi: 10.3758/s13421-025-01785-y (PMC13133198; doi:10.3758/s13421-025-01785-y)
Supplement: Supplementary file 2 — Supplementary file2 (DOCX 31 KB) [file 13421_2025_1785_MOESM2_ESM.docx]

**Supplementary File**

**Calculating variable-specific effect sizes**

In order to make it easier to understand the magnitude of the effects of each variable in the linear mixed model at a glance, we performed an analysis using the *partR2* package (Stoffel, Nakagawa, & Schielzeth, 2017) in R. Notably, however, this package has difficulties in estimating effect sizes that include higher-order interactions, as in this study. Therefore, we decided not to include this analysis in the article and to report it in this file instead. Here, we report a table with an additional column for R-squared values added to Tables 4 and 5 published in the article. These effect sizes provide useful information, but should be interpreted with caution. Note that the analyses for retrieval latency (Table 6), specific memory response (Table S1), categoric memory response (Table S2) did not converge, possibly due to the complexity of the model.

**Reference**

Stoffel, M. A., Nakagawa, S. & Schielzeth, H. (2017). rptR: Repeatability estimation and variance decomposition by generalized linear mixed-effects models. *Methods in Ecology and Evolution*, *8*, 1639-1644.
