# Supplementary material for: Autobiographical memory retrieval in the context of self-schema updating: Does specific recall have power?
Source: Mem Cognit. 2025 Aug 27;54(3):865–82. doi: 10.3758/s13421-025-01785-y (PMC13133198; doi:10.3758/s13421-025-01785-y)
Supplement: Supplementary file 2 [file 13421_2025_1785_MOESM1_ESM.docx]

Table S1. Generalized linear mixed model for specific memory recall

Predictors	*Odds Ratios*	*95%CI*	*p*
(Intercept)	0.51	0.36 – 0.72	**<0.001**
Cue valence	0.97	0.75 – 1.26	.831
Self/Other	0.82	0.64 – 1.06	.136
Consistency	1.46	1.13 – 1.89	**.004**
BDI-II	1.01	0.98 – 1.03	.670
Cue valence * Self/Other	1.05	0.63 – 1.76	.851
Cue valence * Consistency	1.74	1.04 – 2.91	**.035**
Self/Other * Consistency	1.03	0.61 – 1.72	.920
Cue valence * BDI-II	1.01	0.99 – 1.02	.500
Self/Other * BDI-II	1.00	0.98 – 1.02	.826
Consistency * BDI-II	0.99	0.97 – 1.01	.167
Cue valence * Self/Other * Consistency	0.93	0.33 – 2.61	.896
Cue valence * Self/Other * BDI-II	1.00	0.96 – 1.03	.931
Cue valence * Consistency * BDI-II	0.98	0.95 – 1.02	.265
Self/Other * Consistency * BDI-II	1.00	0.96 – 1.04	.942
Cue valence * Self/Other * Consistency * BDI-II	0.96	0.89 – 1.03	.281
**Random Effects**			
σ^2	3.29		
$\tau_{00\ \mathrm{ParticipantID}}$	0.67		
ICC	0.17		
$N_{\mathrm{ParticipantID}}$	100		
Observations	4800		
Marginal R^2 / Conditional R^2	0.009 / 0.176		

Table S2. Generalized linear mixed model for categoric memory recall

Predictors	*Odds Ratios*	*95%CI*	*p*
(Intercept)	0.27	0.17 – 0.41	**< .001**
Cue valence	1.29	0.99 – 1.69	.061
Self/Other	1.65	1.26 – 2.15	**< .001**
Consistency	1.35	1.03 – 1.76	**.029**
BDI-II	1.02	0.99 – 1.05	.183
Cue valence * Self/Other	0.83	0.49 – 1.42	.499
Cue valence * Consistency	1.44	0.84 – 2.46	.180
Self/Other * Consistency	0.85	0.50 – 1.45	.549
Cue valence * BDI-II	0.98	0.97 – 1.00	.085
Self/Other * BDI-II	0.99	0.97 – 1.01	.349
Consistency * BDI-II	0.99	0.97 – 1.01	.333
Cue valence * Self/Other * Consistency	0.37	0.13 – 1.09	.071
Cue valence * Self/Other * BDI-II	1.00	0.97 – 1.04	.833
Cue valence * Consistency * BDI-II	0.99	0.96 – 1.03	.588
Self/Other * Consistency * BDI-II	1.01	0.98 – 1.05	.545
Cue valence * Self/Other * Consistency * BDI-II	1.02	0.95 – 1.10	.560
**Random Effects**			
σ^2	3.29		
$\tau_{00\ \mathrm{ParticipantID}}$	0.99		
ICC	0.23		
$N_{\mathrm{ParticipantID}}$	100		
Observations	4800		
Marginal R^2 / Conditional R^2	0.020 / 0.247		

Table S3. Linear mixed model for changes in self-schemas

Predictors	*Estimates*	*95%CI*	*p*
(Intercept)	-0.09	-0.23 – 0.04	.183
Cue valence	0.01	-0.18 – 0.21	.896
Self/Other	0.00	-0.19 – 0.20	.999
Consistency	0.26	0.07 – 0.46	**.009**
BDI-II	0.00	-0.01 – 0.01	.842
Cue valence * Self/Other	-0.44	-0.83 – -0.05	**.027**
Cue valence * Consistency	0.16	-0.23 – 0.55	.423
Self/Other * Consistency	1.64	1.25 – 2.03	**< .001**
Cue valence * BDI-II	0.00	-0.01 – 0.01	.824
Self/Other * BDI-II	0.00	-0.01 – 0.01	.883
Consistency * BDI-II	0.01	-0.01 – 0.02	.259
Cue valence * Self/Other * Consistency	-0.51	-1.29 – 0.27	.196
Cue valence * Self/Other * BDI-II	0.02	-0.00 – 0.05	.091
Cue valence * Consistency * BDI-II	0.00	-0.03 – 0.02	.881
Self/Other * Consistency * BDI-II	0.00	-0.03 – 0.02	.875
Cue valence * Self/Other * Consistency * BDI-II	0.02	-0.03 – 0.07	.487
**Random Effects**			
σ^2	1.74		
$\tau_{00\ ParticipantID}$	0.05		
$\tau_{00\ TrialID}$	0.01		
ICC	0.03		
$N_{ParticipantID}$	100		
$N_{TrialID}$	48		
Observations	2893		
Marginal R^2 / Conditional R^2	0.103 / 0.131		

Table S4. Linear mixed model for changes in self-schemas

Predictors	*Estimates*	*95%CI*	*p*
(Intercept)	-1.32	-1.69 – -0.95	**<0.001**
Retrieval latency	0.29	0.13 – 0.44	**< .001**
Cue valence	0.67	-0.06 – 1.40	.074
Consistency	1.92	1.45 – 2.39	**< .001**
Retrieval latency * Cue valence	-0.29	-0.60 – 0.01	.059
Retrieval latency * Consistency	-0.33	-0.53 – -0.13	**.001**
Cue valence * Consistency	-0.75	-1.69 – 0.18	.114
Retrieval latency * Cue valence * Consistency	0.31	-0.09 – 0.71	.125
**Random Effects**			
σ^2	1.89		
$\tau_{00\ \mathrm{ParticipantID}}$	0.03		
ICC	0.01		
$N_{\mathrm{ParticipantID}}$	101		
Observations	1526		
Marginal R^2 / Conditional R^2	0.157 / 0.169		
